# Signal transduction and modulating pathways in tryptamine-evoked vasopressor responses of the rat isolated perfused mesenteric bed

**DOI:** 10.1016/j.vph.2012.10.007

**Published:** 2013-01

**Authors:** M. Akhtar Anwar, William R. Ford, Amy A. Herbert, Kenneth J. Broadley

**Affiliations:** Division of Pharmacology, Cardiff School of Pharmacy & Pharmaceutical Sciences, Cardiff University, Redwood Building, King Edward VII Avenue, Cathays Park, Cardiff CF10 3NB, UK

**Keywords:** COX, cyclooxygenase, DAG, diacylglycerol, DRC, dose–response curve, 5-HT, 5-hydroxytryptamine, IP, inositol phosphate, IP_3_, inositol 1,4,5-trisphosphate, MAO, monoamine oxidase, l-NAME, N^ω^-nitro-l-arginine methyl ester, NO, nitric oxide, NOS, nitric oxide synthase, PAF, platelet activating factor, PIP_2_, phosphatidylinositol 4,5-bisphosphate, PLA_2_, phospholipase A_2_, PLC, phospholipase C, PKC, protein kinase C, TAAR, trace amine-associated receptor, TxA_2_, thromboxane A_2_, Tryptamine, Rat mesenteric artery, Vasoconstriction, 5-HT_2A_ receptors, Nitric oxide

## Abstract

Tryptamine is an endogenous and dietary indoleamine-based trace amine implicated in cardiovascular pathologies, including hypertension, migraine and myocardial infarction. This study aimed at identifying the signalling pathways for the vasoconstrictor response to tryptamine in rat isolated perfused mesenteric arterial beds and co-released vasodilator modulators of tryptamine-mediated vasoconstriction. Tryptamine caused concentration-dependent vasoconstriction of the mesenteric bed, measured as increases in perfusion pressure. These were inhibited by the 5-HT_2A_ receptor antagonist, ritanserin, indicating mediation via 5-HT_2A_ receptors. The response was inhibited by the phospholipase C (PLC) and phospholipase A_2_ (iPLA_2_) inhibitors, U-73122 and PACOCF_3_, suggesting involvement of phospholipase pathways. Activation of these pathways by tryptamine releases cyclooxygenase (COX) products since indomethacin (non-selective inhibitor of COX-1/2) and nimesulide (selective COX-2 inhibitor) reduced the vasoconstriction. The most likely COX vasoconstrictor product was prostaglandin PGE_2_ since the responses to tryptamine were reduced by AH-6809, a non-selective EP_1_ receptor antagonist. Involvement of the Rho-kinase pathway in the tryptamine-evoked vasoconstriction was also indicated by its reduction by the Rho-kinase inhibitors, Y-27,632 and fasudil. The tryptamine vasoconstriction is modulated by the co-released endothelial vasodilator, nitric oxide. Thus, circulating tryptamine can regulate mesenteric blood flow through a cascade of signalling pathways secondary to stimulation of 5-HT_2A_ receptors.

## Graphical abstract

**Figure:**
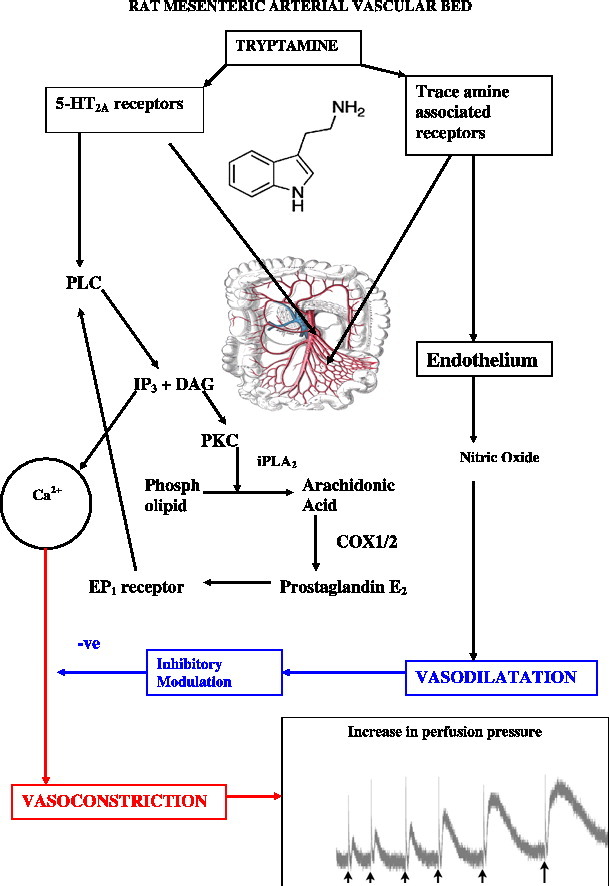

## 1 Introduction

Tryptamine is a biogenic amine structurally related to 5-hydroxytryptamine (5-HT) that is generated in the body by neural and peripheral tissues. It is also formed by the microflora in the gastrointestinal tract and is a component of many food items. Tryptamine is implicated in various cardiovascular pathologies, including hypertension, myocardial infarction and migraine. Migraine cluster headaches have been shown to be relieved by psilocybin, a component of magic mushrooms, whose active metabolite, psilocin is a trypamine analogue (N,N-dimethyltryptamine). This action is at a subhallucinogenic dose and likely due to cardiovascular actions (Sewell et al., 2006). The concentration of tryptamine in serum is correlated with that of its precursor l-tryptophan (Wollman et al., 1985), which is metabolised into tryptamine by aromatic l-amino acid decarboxylase. Tryptamine is deaminated by monoamine oxidase (MAO) types A and B (Tipton et al., 2004) to indole-3-acetaldehyde, which is subsequently reduced by aldehyde dehydrogenase to indole-3-acetic acid (Weissbach et al., 1959).

Tryptamine increases blood pressure (Eble, 1965), an effect that has long been held to be due to indirect sympathomimetic actions, since it may be regarded as a trace amine (Zucchi et al., 2006). Indirect sympathomimetic amines release noradrenaline from sympathetic neurones onto vascular α-adrenoceptors to cause vasoconstriction (Trendelenburg, 1972). However, in isolated vascular tissues, tryptamine has been shown to cause vasoconstriction of rabbit aorta not by an indirect mechanism but by direct stimulation of both α-adrenoceptors and 5-HT receptors (Stollak and Furchgott, 1983). Vasoconstriction by tryptamine has also been demonstrated in rat mesenteric arteries (Watts et al., 1994), rat caudal arteries (Hicks and Langer, 1983, Bradley et al., 1985) and rat aorta (Fehler et al., 2010). Hicks and Langer (1983) suggested that specific tryptaminergic receptors mediated the vasoconstriction by tryptamine in rat tail arteries. More recently, the vasoconstriction of rat aorta by tryptamine and other trace amines has been shown to be resistant to blockade by α-adrenoceptor and 5-HT antagonists (Broadley et al., 2009, Fehler et al., 2010). The vasoconstrictor response was attributed (Broadley et al., 2009, Broadley, 2010) to the recently described trace amine-associated receptors (TAARs) (Borowsky et al., 2001, Bunzow et al., 2001).

Given the importance of both dietary and endogenous tryptamine in health and disease, there is a paucity of data on the receptors activated by this amine and their signalling pathways in vascular smooth muscle. The few mechanistic studies that do exist have focussed on the phospholipids. In rat cortical slices, tryptamine-induced stimulation of inositol phosphate (IP) accumulation was insensitive to atropine, cyproheptadine, haloperidol, phenoxybenzamine and propranolol indicating that classical neurotransmitter receptors were not involved (Osborne et al., 1986). In a later study, tryptamine activation of primary cultures of rat cerebellar granule cells increased IP turnover, which was not counteracted by atropine, ketanserin and prazosin (Ishitani et al., 1994). Subsequently, it was shown that in NIH3T3 fibroblasts stably expressing the 5-HT_2A_ receptor, tryptamine activated the phospholipase C (PLC) and phospholipase A_2_ (PLA_2_) signalling pathways (Kurrasch-Orbaugh et al., 2003).

In the rat mesentery, we have recently demonstrated that tryptamine mediates both vasopressor and vasodepressor responses. The vasoconstrictor response was blocked by the 5-HT antagonists, ritanserin and ketanserin, and is therefore mediated predominantly via 5-HT_2A_ receptors (Anwar et al., 2012). However, there is a clear lack of data on the mechanisms of tryptamine-induced changes in vascular tone, specifically in the resistance size arteries of the mesentery. Based on the knowledge from our mesenteric arterial network studies and the above-mentioned cellular and tissue experiments, we undertook the present investigation to assess the contributions made by selected contractile transduction pathways in tryptamine-derived vascular tone. We also determined the possible roles of co-released vasodepressor transducers in modulating tryptamine-evoked vasoconstriction.

Preliminary accounts of some of these findings have been reported to the British Pharmacological Society (Anwar et al., 2006) and the European Microcirculation Society (Anwar et al., 2008).

## 2 Materials and methods

### 2.1 Animal care

Male Sprague–Dawley rats (250–350 g body weight; Harlan, Bicester, Oxfordshire, U.K.) were housed in temperature (22 ± 1 °C) and humidity (50%) controlled quarters on a 12 h light–dark cycle (07.00–19.00 h light and 19.00–07.00 h dark), 4 animals to a cage, and provided with food and water ad libitum. They were killed by cervical dislocation following stunning in accordance with the Home Office Guidance on the operation of The Animals (Scientific Procedures) Act 1986 (H.M.S.O.), and after local ethical review by the Animal Care and Use Committee of Cardiff University.

### 2.2 Isolated mesenteric arterial bed

The mesenteric vascular bed was exposed through a midline laparotomy incision; the superior mesenteric artery was cleaned of adipose and connective tissues. The artery was cannulated, close to the junction with the abdominal aorta, with a PE-50 polyethylene (BD Intramedic, Oxford, U.K.) cannula, which was ligated and secured with cotton ties, and the mesenteric vascular bed was immediately perfused with Krebs'–Henseleit-bicarbonate solution, composition in mM: NaCl 118.0, KC1 4.7, NaHCO_3_ 25.0, CaCl_2_ 2.5, KH_2_PO_4_ 1.2, MgSO_4_ 1.2, and glucose 11 (osmolality ≅ 292 mosmol/kg H_2_O), warmed to 37 °C and gassed continuously with 5% CO_2_/95% O_2_ to maintain the pH at 7.4. The initial flow rate was set at 2 ml per minute using a Gilson peristaltic pump to remove blood and metabolites. Simultaneously, the mesenteric bed was excised by gently separating from the gastrointestinal tract (stomach to anterior of rectum), placed in a thermostatic perfusion chamber, and the arterial network was perfused at a constant flow rate (4 ml min^− 1^) that was maintained throughout the experimental procedure (McGregor, 1965). Changes in perfusion pressure, a reflection of vascular resistance, were monitored by a pressure transducer (Elcomatic EM 750, Elcomatic Ltd., Glasgow, U.K.) located immediately proximal to the inflow cannula. The transducer was coupled to a PowerLab/4SP computerised data acquisition system (AD Instruments, Charlgrove, Oxon, U.K.) and Chart version 5 software (AD Instruments, U.K.) to display and analyse data. A bubble trap, distal to the cannulated mesentery and proximal to the perfusate solution, removed any air bubbles in the perfusate and also dampened pulses in flow.

### 2.3 Experimental protocols

After an equilibration period of 1 h, each arterial preparation was subjected to one of the following experimental protocols. Concentrations of signalling pathway inhibitors were chosen based on previously published data. Dose–response curves (DRC) to tryptamine by bolus injection (100 μl volume) were constructed in logarithmic increments in the absence and repeated in the presence of continuous infusion of inhibitors in the same preparation. The response of the preceding dose was permitted to return to the base line before the start of the next incremental dose. Each inhibitor was infused for approximately 20 min prior to the commencement of the subsequent DRC. A 30 min washout interval was allowed between successive dose–response curves.

#### 2.3.1 Effects of shear stress

Shear stress, the frictional force generated by blood flow acting on the luminal wall, is known to have a profound effect on the endothelium, and therefore its influence on vascular reactivity (Davies, 1995). Consequently, flow–pressure relationships were determined by incrementally ramping flow rate to achieve a perfusion pressure of 45 mm Hg (Fulep et al., 2002). The flow rate–perfusion pressure relationship was examined in the absence and presence of l-NAME (100 μM).

#### 2.3.2 Drug interventions

To elucidate whether the endothelium could modulate tryptamine-associated vasoconstriction, endothelium denudation was achieved by perfusing distilled water for 4 min through the mesenteric arterial network, followed by perfusion with Kreb's solution for 30 min. The extent of endothelium disruption was confirmed by acetylcholine-induced (10^− 7^ M) relaxation of pre-constricted vascular bed (10 μM phenylephrine). Tissues exhibiting inhibition of the acetylcholine-induced vasodilatation by more than 50% were considered acceptable for inclusion in the study.

To examine the roles of 5-HT_2A_ receptors, nitric oxide (NO), monoamine transporters, monoamine oxidases A and B (MAOA/B), phospholipase C (PLC), phospholipase A_2_ (PLA_2_), Rho-kinases (ROCK), cyclooxygenases 1 and 2 (COX1 and COX2), prostanoid receptors (EP_1_ and TP) and prostacyclin synthase (PGI_2_ synthase), DRCs for tryptamine were constructed in the absence and presence of ritanserin (100 pM), l-NAME (100 μM), cocaine (10 μM), U73122 (synthetic aminosteroid compound, 10 μM), PACOCF_3_ (calcium-independent PLA_2_ antagonist, 10 μM); indomethacin (a non-selective (COX) inhibitor, 10 μM), nimesulide (selective COX-2 antagonist, 10 μM), tranylcypromine (a prostacyclin synthase and non-specific MAOA and MAOB inhibitor, 10 μM), AH 6809 (PGE_2_ receptor (EP_1_ and EP_2_)/less selective PGD_2_ receptor (DP) antagonist, 10 μM), ICI 192,605 (TP receptor antagonist; 10 μM), and the ROCK inhibitors, Y-27,632 (10 μM) and fasudil (also known as HA-1077, 20 μM).

### 2.4 Data and statistical analysis

Responses to each dose of tryptamine were measured as the increase in perfusion pressure from the baseline immediately preceding the first dose. Data are expressed as mean ± S.E.M. n indicates the number of animals used. Individual DRCs were plotted as mean increase in perfusion pressure (mm Hg), and the dose–response curves were fitted to a four parameter logistic model to calculate ED_50_ values (the concentration of agonist which produces a response halfway between the baseline and maximum response, E_max_) using FigP (Biosoft, Cambridge, U.K.). From these quantities, geometric means of ED_50_ and E_max_ with 95% confidence limits were computed.

Linear correlation analysis and significances of differences between control and paired E_max_ and ED_50_ values were obtained by paired Student's *t*-test. Comparisons of E_max_ and ED_50_ values between different tissues were made by Student's unpaired *t*-test, and comparisons between more than two groups were made by ANOVA followed by Tukey's multiple comparison test. A *P* value of < 0.05 was considered to be statistically significant.

### 2.5 Drugs and chemicals

The following drugs were used and were purchased from Tocris (Bristol, U.K.): AH-6809, fasudil, 1-[6-[[(17b)-3-methoxyestra-1,3,5(10)-trie-17-yl]amino]hexyl]-1H-pyrrole-2,5-dione, ICI-192,605 (4-(Z)-6-(2-o-Chlorophenyl-4-o-hydroxyphenyl-1,3-dioxan-cis-5-yl)hexenoic acid), nimesulide, PACOCF_3_ (palmitoyl trifluoromethyl ketone), ritanserin, U73122 and Y-27,632. The following drugs were acquired from Sigma-Aldrich (Poole, UK): acetylcholine, cocaine hydrochloride, 5-hydroxytryptamine (5-HT) hydrochloride, indomethacin [1-(4-chlorobenzoyl)-5-methoxy-2-methyl-1H-indole-3-acetic acid], l-NAME (N_ω_-nitro-l-arginine methyl ester), pargyline, tranylcypromine (trans-2-phenyl-cyclopropylamine hydrochloride), tryptamine hydrochloride, U-46619 (9,11-dideoxy-9a, 11a-methanoepoxy prostaglandin F2a).

All agonists and inhibitors were prepared in distilled water, except indomethacin, U73122, ICI 192,605, ritanserin and PACOCF_3_ which were dissolved in ethanol and AH6809 which was dissolved in 1.1 eq of NaOH. The stock solutions were stored frozen in aliquots, and when required were thawed and diluted. All drug dilutions were made using Krebs' solution. To eliminate any possible effect of the vehicle on vascular reactivity, the concentration of ethanol used when required was ≤ 0.1% (vol/vol) in the perfusion fluid (Moreau et al., 1997).

## 3 Results

Basal perfusion pressure was 21.2 ± 0.5 mm Hg for n = 75 animals; unless otherwise indicated, there was no effect of inhibitors on basal perfusion pressure.

### 3.1 Responses to tryptamine

**Fig. 1:**
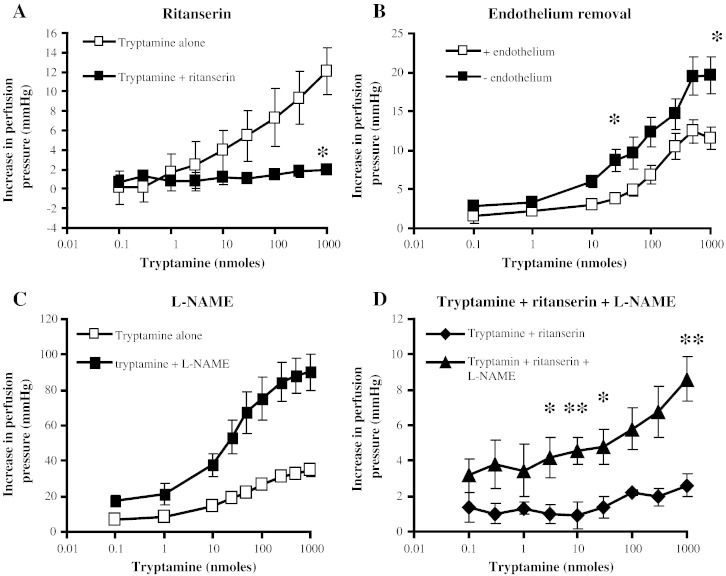
Dose–response curves for increases in perfusion pressure by tryptamine of rat isolated perfused mesenteric arterial bed. Doses of tryptamine were administered as individual boluses (nmoles/100 μl). Each response is the mean ± S.E.M. increase in perfusion pressure. A. Dose–response curves for vasoconstrictor responses to tryptamine in the absence (□) and repeated in the presence (■, n = 3) of ritanserin (100 pM). * Significantly different from the absence of ritanserin, *P* < 0.05. B. Dose–response curves for tryptamine with intact endothelium (□, n = 4) and in de-endothelialised (■, n = 4) mesenteric arterial bed. * Significantly different from intact endothelium, *P* < 0.05. C. Dose–response curves in the absence (□) and presence (■, n = 7) of l-NAME (100 μM). All points significantly different between tryptamine alone and with l-NAME, *P* < 0.05. D. Dose–response curves in the presence of ritanserin (◆, 100 pM) and in the additional presence (▲, n = 3) of l-NAME (100 μM). * Significantly greater than ritanserin alone, *P* < 0.05, ** P < 0.01.

Tryptamine caused dose-related increases in perfusion pressure. These responses were inhibited in the presence of ritanserin (100 pM) (Fig. 1A).

### 3.2 De-endothelialization and inhibition of nitric oxide synthase (NOS)

On denudation of the mesenteric arteries, the basal perfusion pressure was significantly increased (13 ± 1 vs 18 ± 1 mm Hg, *P* < 0.01). Moreover, the removal of endothelium augmented the maximum contractile response to tryptamine (Fig. 1B). In the presence of l-NAME (100 μM) the constrictor responses to tryptamine were potentiated (Fig. 1C) and the maximum response was significantly greater than in the control mesenteries (Table 1). When l-NAME was introduced in the presence of ritanserin (100 pM), small vasoconstrictor responses to tryptamine were reinstated (Fig. 1D).

**Table 1:** Potency and maximum vasoconstrictor responses for tryptamine in the absence and presence of inhibitors in the rat isolated perfused mesenteric arterial beds.

Signalling pathway and inhibitor	Potency (ED_50_, nmol/100 μl)	Maximum effects (E_max_, mm Hg)	n
5-HT_2A_ receptors			
Control	32.0 (22.4–45.6)	37.3 ± 3.3	9
Ritanserin (100 pM)	103.9 (14.2–760)	2.0 ± 0.4^$$^	3
Nitric oxide synthase			
Control	35.2 (25.9–47.8)	32 ± 3	7
l-NAME (100 μM)	33.4 (17.4–64.2)	90.1 ± 10.3^⁎⁎⁎^	7
l-NAME + ritanserin	9.0 (1.3–61.0)	8.6 ± 1.2	3
Denudation			
+ Endothelium	81.0 (63.3–103.5)	12.4 ± 1.5	4
− Endothelium	96.8 (24.7–379.2)	20.0 ± 2.3^⁎^	4
Monoamine transporter			
Control	46.9 (33.7–65.4)	27 ± 1	4
Cocaine (10^− 5^ M)	46.4 (33.2–64. 7)	51 ± 3^⁎⁎^	4
Cocaine + l-NAME	22.1 (6.5–75.5)^##^	126.4 ± 6.9^###^	4
Phospholipase C			
Control	38.8 (24.1–62.5)	43 ± 6	4
U73,122 (10^− 5^ M)	ND	8.6 ± 1.7^⁎⁎^	4
Phospholipase A_2_			
Control	29.6 (12.8–68.4)	18 ± 2	3
PACOCF_3_ (10^− 5^ M)	92.1 (55.5–152.8)^⁎^	18 ± 2	3
Rho-kinase			
Control	26.2 (12.5–54.7)	20 ± 4	3
Y-27,632 (10^− 5^ M)	18.9 (5.9–61.0)	11 ± 3^⁎⁎^	3
Control	27.50 (13.24–57.1)	26 ± 3	3
Fasudil (2 × 10^− 5^ M)	18.9 (4.3–82.6)	9 ± 1^⁎⁎^	3
COX-1 and COX-2			
Control	29.2 (17.0–50.1)	25 ± 3	5
Indomethacin (10^− 5^ M)	ND	10.4 ± 1.7^⁎⁎^	5
COX-2			
Control	28.9 (12.7–65.7)	45 ± 1	3
Nimesulide (10^− 5^ M)	57.6 (52.7–63.0)^⁎^	22 ± 2^⁎⁎^	3
Prostanoid EP_1_ receptor			
Control	32.0 (21.1–48.7)	20 ± 3	4
AH-6809 (10^− 5^ M)	49.0 (32.6–73.7)*	14 ± 3	4
Prostacyclin synthase/MAO			
Control	29.4 (21.2–40.8)	28 ± 7	5
Tranylcypromine (10^− 5^ M)	20.1 (14.1–28.6)^⁎^	27 ± 3	5
Thromboxane TP receptor			
Control	29.8 (20.5–43.4)	24 ± 5	4
ICI 192605 (10^− 6^ M)	28.8 (15.6–53.5)	27 ± 5	4

Potency is represented as the geometric mean (with 95% confidence intervals) ED_50_ (nmole/100 μl) and the maximum response is displayed as arithmetic mean ± S.E.M maximum increase in perfusion pressure (mm Hg). n is the number of animals. * Represents significant differences from paired control values by Student's paired *t*-test, *P* < 0.05, ** *P* < 0.01 and *** *P* < 0.001. ^$$^ Significant difference from unpaired controls by Student's unpaired *t*-test, *P* < 0.01. ^##^ Significant differences between the l-NAME plus cocaine group and control or cocaine alone by one-way ANOVA with Tukey's multiple comparison test, *P* < 0.003, ^###^*P* < 0.0001. ND, not determined.

**Fig. 2:**
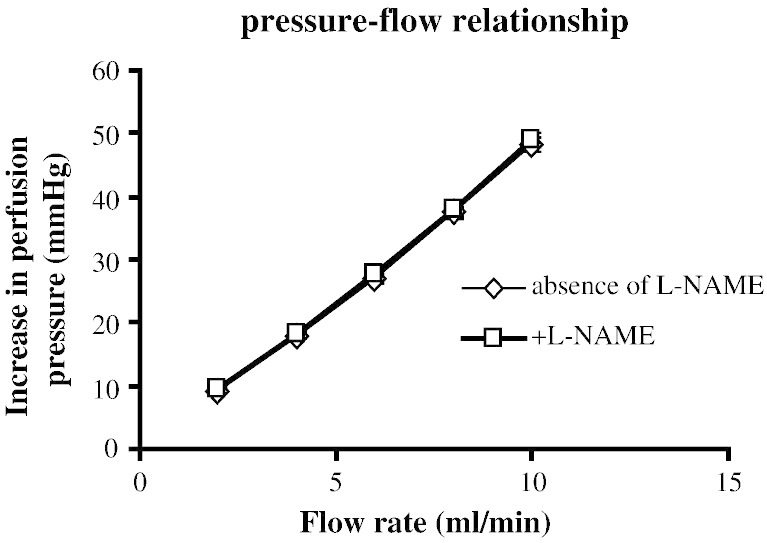
Correlation between flow rate and increase in perfusion pressure in rat isolated perfused mesenteric arterial bed in the absence (⋄, n = 6, y = 4.9 × − 1.32, r^2^ = 0.99) and presence (□, n = 6, y = 4.90 × − 0.86, r^2^ = 0.99) of l-NAME (100 μM).

To examine the role of shear stress on the perfusion pressure and whether nitric oxide was released by the increasing shear stress during vasoconstrictor responses, the relationship between flow rate and perfusion pressure was examined. Increasing flow rate resulted in a linear increase in perfusion pressure (Fig. 2). This flow rate–perfusion pressure relationship was identical in the presence of l-NAME (100 μM) (Fig. 2).

### 3.3 Phospholipase and cyclo-oxygenase pathways

**Fig. 3:**
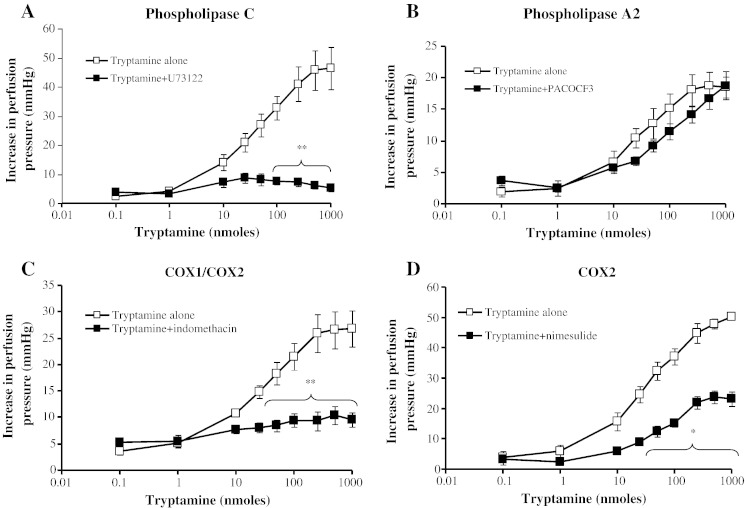
Effects of phospholipase and COX inhibitors on dose–response curves for tryptamine-induced vasoconstriction of rat isolated perfused mesentery. Dose–response curves in the absence (□) and presence (■) of A. phospholipase C inhibitor, U73,122 (10 μM, n = 4), B. phospholipase A_2_ inhibitor, PACOCF_3_ (10 μM, n = 3), C. COX-1/COX-2 inhibitor, indomethacin (10 μM, n = 5), and D. COX-2 inhibitor, nimesulide (10 μM, n = 3). * Significantly different points from tryptamine alone, *P* < 0.05, ** *P* < 0.01. Each response is the mean ± S.E.M. increase in perfusion pressure.

The non-selective phospholipase C inhibitor, U73122 (1 μM), abolished the tryptamine-induced vasoconstrictor responses of the mesenteric arteries (Fig. 3A and Table 1), and it was not possible to ascertain an ED_50_ value. The competitive phospholipase A_2_ (iPLA_2_) inhibitor, PACOCF_3_, also antagonised the responses to tryptamine as a shift of the DRC to the right (Fig. 3B), reflected by the reduced potency (Table 1). Indomethacin (a non-specific COX inhibitor) almost completely abolished the vasoconstrictor responses to tryptamine (Fig. 3C), precluding determination of an ED_50_ value. Nimesulide, a specific COX-2 inhibitor, significantly attenuated the maximum response from 45 ± 1 to 22 ± 2 mm Hg (Fig. 3D).

### 3.4 Prostanoid receptors

**Fig. 4:**
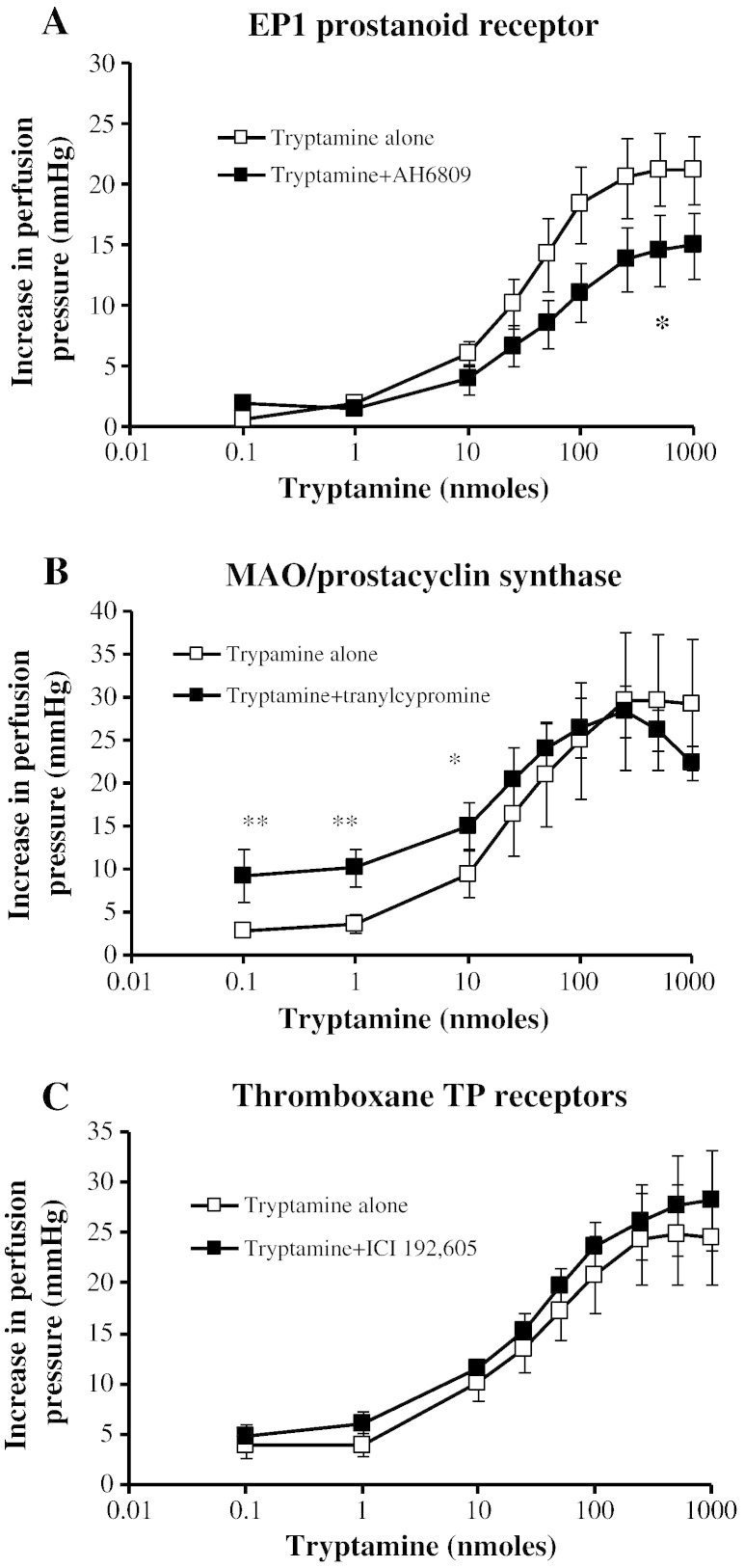
Effects of prostanoid inhibitors on dose–response curves for tryptamine-induced vasoconstriction of rat isolated perfused mesentery. Dose–response curves in the absence (□) and presence (■) of A. prostaglandin EP_1_ receptor antagonist, AH-6809 (10 μM, n = 4), B. prostacyclin synthase/MAO inhibitor, tranylcypromine (10 μM, n = 5), and C. thromboxane TP receptor antagonist, ICI 192,605 (1 μM, n = 4). * Significantly different points from tryptamine alone, *P* < 0.05, ** *P* < 0.01. Each response is the mean ± S.E.M. increase in perfusion pressure.

The EP_1_ prostanoid receptor inhibitor, AH6809 (10 μM), shifted the DRC for tryptamine to the right and significantly increased the ED_50_ value (Fig. 4A). Tranylcypromine (a prostacyclin synthase inhibitor and non-selective MAO-A and MAO-B inhibitor) increased the sensitivity to tryptamine only at the lower concentrations (Fig. 4B). The potency and maximum responses of the mesenteric arterial bed to tryptamine were unaltered in the presence of the thromboxane TP receptor antagonist ICI 192,605 (Fig. 4C).

### 3.5 Rho-kinase inhibitors

**Fig. 5:**
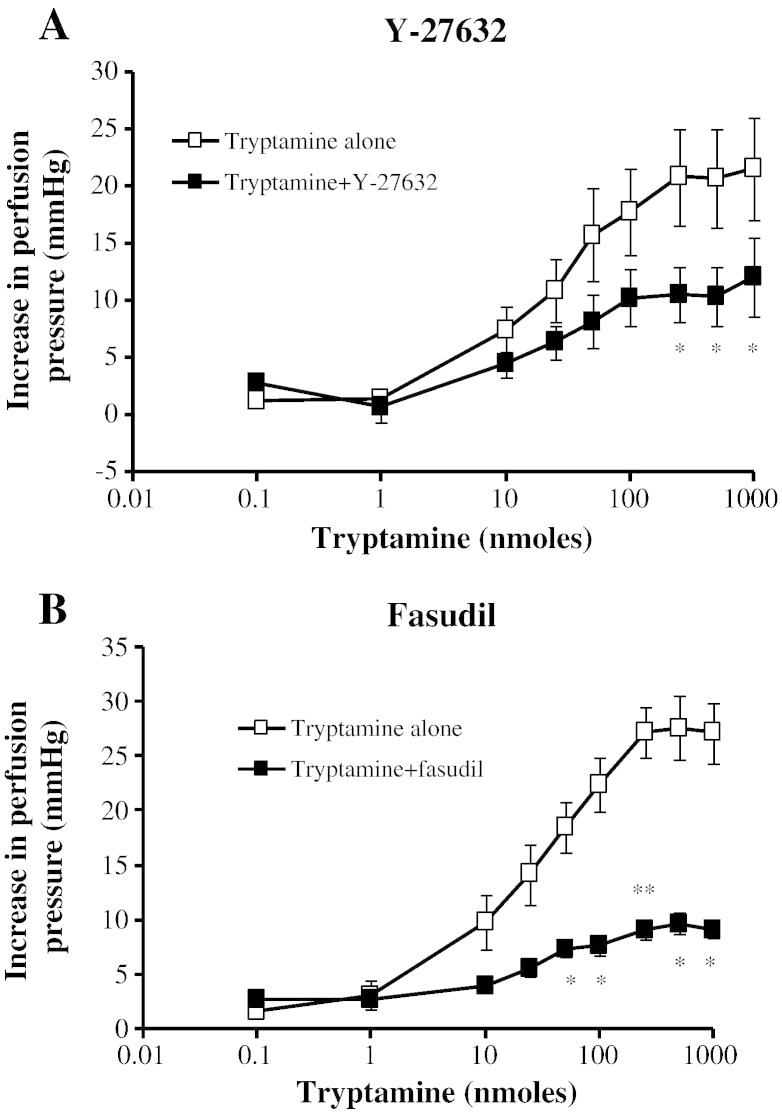
Effects of Rho-kinase inhibitors on dose–response curves for tryptamine-induced vasoconstriction of rat isolated perfused mesentery. Dose–response curves in the absence (□) and presence (■) of A. Y-27632 (10 μM, n = 3) and B. fasudil (HA-1077, 20 μM, n = 3). * Significantly different from tryptamine alone, *P* < 0.05, ** *P* < 0.01. Each response is the mean ± S.E.M. increase in perfusion pressure.

The tryptamine-dependent vasoconstriction was inhibited by the Rho/Rho-kinase inhibitors. The maximum response to tryptamine was significantly inhibited to 45% by Y-27632 (Fig. 5A) and to 65% by fasudil (Fig. 5B).

### 3.6 Monoamine transporter

**Fig. 6:**
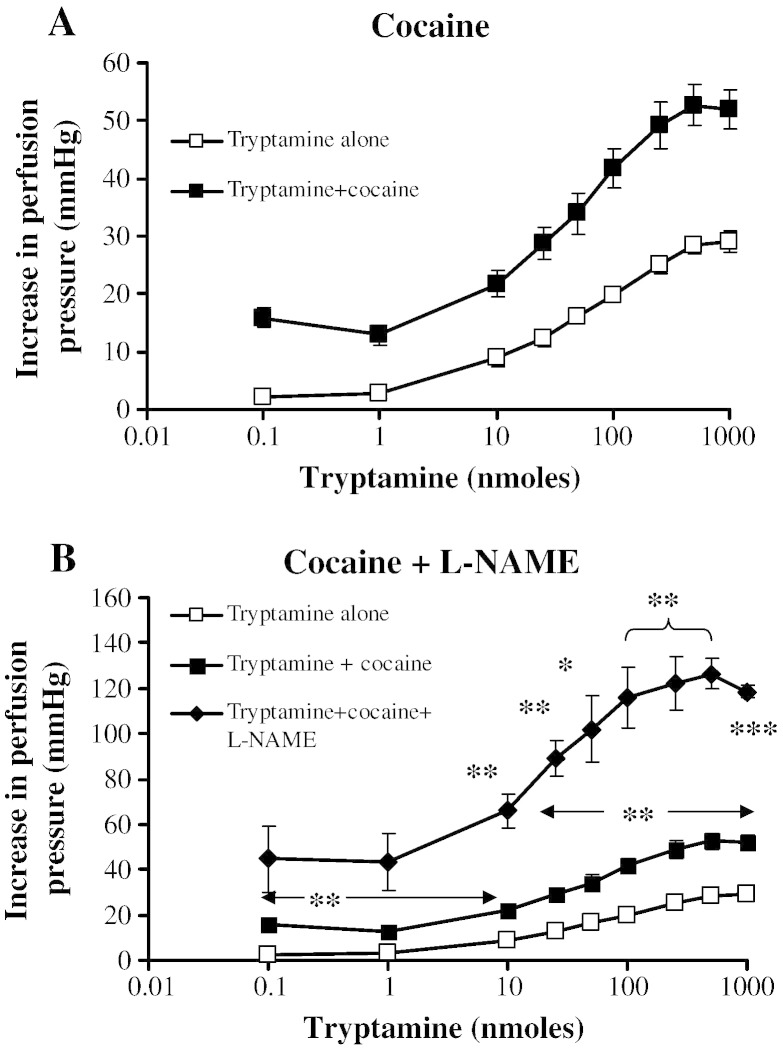
Effects of the monoamine transport inhibitor, cocaine, on dose–response curves for tryptamine-induced vasoconstriction of rat isolated perfused mesentery. Dose–response curves in the absence (□) and presence (■) of cocaine (10 μM, n = 4) and, in separate experiments (B.), in the additional presence (♦) of l-NAME (100 μM, n = 4). All points in A. significantly different between tryptamine alone and with cocaine, *P* < 0.05. In B. * Significantly different from tryptamine alone, *P* < 0.05, ** *P* < 0.01, *** *P* < 0.001. Each response is the mean ± S.E.M. increase in perfusion pressure.

The presence of the neuronal amine transport inhibitor, cocaine (10 μM), significantly enhanced the tryptamine vasoconstrictor responses without altering the sensitivity (Fig. 6A and Table 1). When cocaine (10 μM) and l-NAME (100 μM) were combined, there was an additional potentiation, the maximum vasoconstriction reaching 126.4 ± 6.9 mm Hg, which was significantly greater than with l-NAME alone (90.1 ± 10.3 mm Hg).

## 4 Discussion

This study has confirmed previous reports that tryptamine exerts vasoconstrictor properties in the rat isolated mesenteric arteries (Watts et al., 1994) and in the rat isolated perfused mesenteric arterial bed (Anwar et al., 2012). Tryptamine also causes vasoconstriction in rat (Fehler et al., 2010) and rabbit (Stollak and Furchgott, 1983) aorta and rat caudal arteries (Hicks and Langer, 1983, Bradley et al., 1985). This response would explain the increases in blood pressure observed when trypamine is administered to dogs (Eble, 1965). The tryptamine induced pressor response of the mesenteric bed was inhibited by the 5-HT_2A_ receptor antagonist, ritanserin, indicating that the vasoconstriction is largely via activation of 5-HT_2A_ receptors. This is at variance with the vasoconstriction in other vessels such as the rat aorta where the vasoconstriction by tryptamine is not inhibited by another 5-HT_2A_ receptor antagonist, ketanserin (Fehler et al., 2010), although in the rabbit aorta (Stollak and Furchgott, 1983) and rat tail artery (Bradley et al., 1985), 5-HT antagonists were effective. The main aim of this study was to determine the signalling pathways for this 5-HT_2A_-mediated vasoconstriction by tryptamine by the use of appropriate inhibitors. Secondly, we examined whether the response was modulated through co-activation of relaxant signalling mechanisms.

### 4.1 Contractile transducers

#### 4.1.1 Phospholipid signalling cascades

**Fig. 7:**
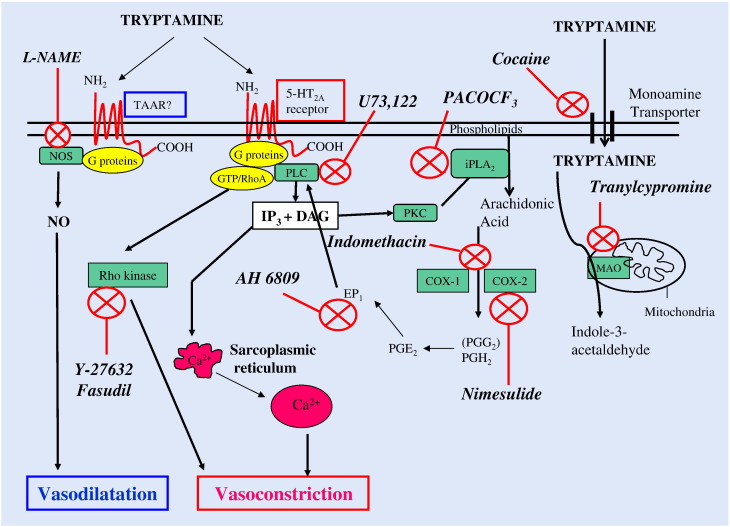
Schematic diagram showing the proposed signal transduction pathways for the vasoconstrictor and vasodilator responses to tryptamine of rat isolated mesenteric vascular bed mediated via 5-HT_2A_ and trace amine-associated receptors (TAAR), respectively. Inhibitors of signalling intermediates are shown in italics and their site of interaction by a red cross ().

Activation of G protein-coupled receptors, such as 5-HT_2A_ receptors, stimulates phospholipase C, the catalysed products of which funnel out to further amplify downstream signalling transducers of smooth muscle contractile responses. Stimulation of phospholipase C catalyses the hydrolysis of the phosphorylated lipid, phosphatidylinositol 4, 5-bisphosphate (PIP_2_) to produce second messengers, inositol 1, 4, 5-trisphosphate (IP_3_) and diacylglycerol (DAG), (Rhee, 2001, Fig. 7). IP_3_ through the activation of IP_3_ receptors (IP_3_Rs), located on store-operated calcium channels, mobilises calcium into the cytosol from intracellular stores (sarcoplasmic/endoplasmic reticulum, Golgi complex and the nuclear envelope), leading to contraction (Berridge, 1993). Tryptamine activates heterotrimeric G protein-coupled 5-HT_2A_ receptors, which are linked to the PLC signalling system since the contractile response was completely eliminated by U73122, a putative blocker of PLC (Osol et al., 1993). DAG, the other transducer arising from PLC activation, stimulates protein kinase C (PKC), (Nishizuka, 1995). DAG activation of PKC is the initial step in the prostaglandin biosynthetic pathway initiated by activation of a family of phospholipase A_2_ (PLA_2_) isozymes [secretory PLA_2_s (sPLA_2_), the cytosolic PLA_2_s (cPLA_2_), calcium-independent PLA_2_s (iPLA_2_) and the platelet activating factor (PAF) acid hydrolases]. These are primarily responsible for agonist-induced hydrolysis of the sn-2 ester bonds in membrane phospholipids, such as phosphotidyl choline and phosphotidylethanolamines, releasing arachidonic acid and lysophospholipids (Schaloske and Dennis, 2006; Fig. 7).

The present investigation has shown that a Ca^2 +^-independent PLA_2_ is involved in tryptamine-induced vasoconstriction, since the response was significantly attenuated by the iPLA_2_ inhibitor, PACOCF_3_. Previous studies have confirmed the iPLA_2_ blocking activity of PACOCF_3_ (Ackerman et al., 1995) and demonstrated the PKC-dependent promoting activity of calcium-independent phospholipase A_2_ β (iPLA_2_β, group VIB PLA_2_) and liberation of arachidonic acid (Jenkins et al., 2002, Akiba and Sato, 2004). Moreover, diacylglycerol has been shown to be a substrate for DAG lipase, which leads to the synthesis of 2-arachidonylglycerol, which is metabolised by monoacylglycerol lipase or fatty acid amidohydrolase to also yield arachidonic acid (Tang et al., 2006).

Interestingly, our results differ from the 5-HT_2A_ receptor-mediated PLC and PLA_2_ signalling pathways in NIH3T3–5HT_2A_ fibroblast cells, which are independently coupled to the receptor (Kurrasch-Orbaugh et al., 2003). This is perhaps due to *a*. overexpression of the receptor in NIH3T3 fibroblasts, *b*. the different cell types involved, or *c*. the static milieu of cell culture as opposed to the dynamic environment of the perfused isolated vascular bed.

#### 4.1.2 Cyclooxygenases

Conversion of arachidonic acid to PGG_2_ is via a cyclooxygenase reaction, which is followed by a peroxidase reaction to PGH_2_, these are the committed steps in prostanoid biosynthesis, and both are mediated by two prostaglandin synthases or cyclooxygenases (COX-1 and COX-2) (Simmons et al., 2004).

Cyclooxygenases play a pivotal role in prostaglandin and/or thromboxane synthesis and their consequent vasoconstrictor effects (Simmons et al., 2004). The present study implicates both COX isoforms as constitutive enzymes in mediating vasoconstriction of the mesenteric arteries by tryptamine. The non-selective COX inhibitor, indomethacin, virtually abolished the vasoconstrictor responses to tryptamine whereas the COX-2-selective inhibitor, nimesulide, only halved the maximum response. This leads to the conclusion that both isoforms are involved in the vasoconstrictor response. Previous reports of expression of the two COX isozymes in different tissues, including the vasculature, from human and animal studies are in accord with our results (Ermert et al., 1998, Wang et al., 2005, Ho and Randall, 2007, Trappe et al., 2008). It is important to note that the abolition of the vasopressor responses by indomethacin indicates that the lipoxygenase and the cytochrome P-450 monoxygenase pathways are not involved in the tryptamine-mediated vasoconstriction.

#### 4.1.3 Prostanoids

The PGH_2_ derived from cyclooxygenase activation is subsequently converted to a variety of bioactive prostanoids, such as thromboxane (TxA_2_) and prostaglandins F_2α_, D_2_, E_2_ and I_2_ (prostacyclin), depending on the downstream enzymatic machinery present in a particular cell type (Coleman et al., 1994, Breyer et al., 2001). Prostaglandin PGE_2_ has been implicated in a plethora of physiological processes in vascular smooth muscle and endothelial cells, including vascular tone, cellular signalling, proliferation, migration and tubulogenesis (Sugimoto and Narumiya, 2007). PGE_2_ exerts the majority of these pleiotropic actions in diverse tissues through a family of four G protein-coupled heptahelical cell surface receptors (EP_1_ to EP_4_), (Narumiya et al., 1999). The EP_1_ receptor antagonist, AH-6809, significantly inhibited the tryptamine-induced vasoconstriction, illustrating that PGE_2_ is probably the main prostanoid generated which induces tryptamine vasoconstriction in the mesenteric arterial bed via EP_1_ receptors. Stimulation of the EP_1_ receptor, through a G_q_ protein, activates PLC/inositol triphosphate and protein kinase C (PKC) signalling and is coupled to intracellular Ca^2 +^ elevation (Boie et al., 1997), resulting in vasoconstriction (Fig. 7). In support of a role for EP_1_ receptors in mediating vasoconstrictor responses, PGE_2_- and 17-phenyl-trinor-PGE_2_ (selective EP_1_ receptor agonist)-induced vasoconstriction in isolated pressurised gracilis muscle arterioles of db/db mice was attenuated by pre-treatment with AH6809 (Rutkai et al., 2009). We next examined whether thromboxanes and thromboxane receptors (TP) were involved in the vasoconstrictor response. The thromboxane receptor inhibitor ICI 192,605 had no effect on the vasoconstrictor responses to tryptamine. Previous studies have shown that vasoconstriction of rat mesenteric arteries by PGE_2_ was unaffected by the thromboxane A_2_ (TxA_2_) receptor blocker SQ 29,548, whereas the response to PGF_2α_ was abolished. The thromboxane A_2_ mimetic, U-46619, also contracted the rat mesenteric resistance arteries but was inhibited by an EP_1_ receptor antagonist (SC-19220) and attributed to release of PGE_2_ by U-46619 into the mesenteric perfusate (Bolla et al., 2004). A link can therefore be established in the vasoconstrictor response to tryptamine between activation of phospholipases C and A_2_, liberation of arachidonic acid and generation of the vasoconstrictor prostaglandin E_2_ via cyclooxygenase. A similar link has been shown in cultures of A-10 vascular smooth muscle cells isolated from wild-type mice, where iPLA_2_β catalysed the liberation of arachidonic acid which binds to COX-2 to produce PGE_2_. In contrast, the concentration of PGE_2_ was dramatically reduced in media obtained from iPLA_2_β-null mice VSMC cultures (Moon et al., 2008). Thus, tryptamine-induced contraction appears to be mediated via the prostanoid PGE_2_ through EP_1_ receptors, but there is no participation of thromboxanes or receptors for TxA_2_.

#### 4.1.4 Rho-kinases

Heterotrimeric G-proteins of the Gα_12_ and Gα_13_ family transduce signals emanating from GPCRs to activate the low molecular weight guanosine triphosphate (GTP)-binding protein RhoA, a member of the Ras family of proteins, and its downstream target, Rho-kinase (a p160 Rho-associated coiled-coil-containing protein kinase, a serine/threonine specific kinase). Rho-kinase has 2 isoforms: ROKα/ROCKII and ROKβ/ROCKI, which are important regulators of vascular tone (Somlyo and Somlyo, 2003). During this calcium-independent process, Rho-kinase causes inhibition of myosin light chain phosphatase activity by phosphorylation of its myosin-binding subunit (a regulatory domain), resulting in elevated vascular tension. This phenomenon is referred to as calcium sensitisation (Uehata et al., 1997).

We have demonstrated that the 5-HT_2A_ receptors stimulated by tryptamine are coupled to the Rho/ROCK signalling pathway by way of attenuation of the vasoconstrictor response to tryptamine by fasudil (active component: hydroxyfasudil) and Y-27632. Fasudil and Y-27632 block the activity of Rho-kinase by competing with the ATP-binding site on the enzyme (Jacobs et al., 2006).

### 4.2 Dilator mediators

#### 4.2.1 Nitric oxide

Inhibition of NO release with the nitric oxide synthase inhibitor, l-NAME, caused almost three-fold increase in the maximum vasoconstriction of the rat mesentery by tryptamine. Similarly, endothelium denudation potentiated the maximum response. The heterotrimeric G protein Gα_12_ is coupled to eNOS leading to elevated intracellular concentrations of eNOS (Andreeva et al., 2006). The effects of NO are mediated primarily through the direct activation of soluble guanylate cyclase, generating cyclic guanosine monophosphate (cGMP) (Andreopoulos and Papapetropoulos, 2000, Lucas et al., 2000), which stimulates protein kinase G (PKG, also termed cGMP-targeted kinase, cGK), which can suppress Gα_q_ stimulation by interaction with regulator of G-protein coupled signalling 2 (RGS2) (Hofmann et al., 2000). To note, vasodilation of mice aortic rings by PGE_2_ interaction with the EP_4_ receptor results in NO formation (Hristovska et al., 2007). The high bioavailability of NO counteracts the contractile actions of COX-metabolites (Miyamoto et al., 2007) and Rho-kinase (Sauzeau et al., 2000). Consequently, NO serves as a homeostatic buffer against incremental or excessive vasoconstriction and smoothes out excessive fluctuations in blood pressure.

When NO was inhibited by l-NAME after blockade of the tryptamine vasoconstriction by the 5-HT_2A_ antagonist, ritanserin, small vasoconstrictor responses were restored. These cannot be due to 5-HT_2A_ receptor stimulation overcoming the ritanserin blockade as the doses of tryptamine are unchanged. Our previous study showed that in the presence of 5-HT_2A_ receptor blockade and with perfusion pressure raised by perfusion with phenylephrine, tryptamine causes dose-related vasodilatation which is NO-mediated (Anwar et al., 2012). When this vasodilator action is removed by l-NAME, a vasoconstriction appears. This was not examined further but may be due to activation of trace amine-associated receptors, which appear to mediate vasoconstrictor responses to tryptamine and other trace amines in other blood vessels such as coronary arteries (Herbert et al., 2008) and rat aorta (Fehler et al., 2010).

#### 4.2.2 Shear stress

According to the Hagen–Poiseuille equation, vascular resistance is a function of vascular geometry (radius and length of vessel) and viscosity of fluid (η). The resistance of a blood vessel is related to the inverse of the fourth power of vessel diameter and therefore small reductions in diameter have significant consequences for vascular resistance. Graded increases in flow rate through the rat mesenteric bed induced by raising the pump flow rate were associated with corresponding rises in perfusion pressure. This is a reflection of an increase in vascular smooth muscle tone due to elevations in wall shear stresses, which leads to an increase in vascular resistance. The possibility was considered that these increases in shear stress might cause release of vasodilator NO which could dampen the pressure increases. However, inhibition of NO synthesis with l-NAME had no influence on flow rate–perfusion pressure relationship. Our results are consistent with the findings of unchanged perfusion pressure after incubation with l-NAME in the non-pregnant rat isolated uterine bed (Fulep et al., 2002) and non-pregnant rat isolated perfused mesenteric arteries (Cockell and Poston, 1996). However, they contrast with flow experiments on cultured endothelial cells (Kuchan and Frangos, 1994), where NO was found to be released. Therefore, in our study NO release can be attributed to tryptamine stimulation of post-receptor pathways and not as a result of any shear forces exerted on the luminal wall by the vasoconstriction. The possibility, however, arises that increasing perfusion pressure by pump-mediated increases in flow does not entirely mimic the increase due to vasoconstriction. The possibility must be considered that a part of the NO release by tryptamine results from conformational changes of endothelial cells arising from vasoconstriction. The effects of l-NAME on a wider range of vasoconstrictor agents acting via different receptors would be required to test this idea further.

It is worth mentioning that links have been established between nitric oxide and the Rho-kinase signal transduction pathways. NO can cause vasodilatation through inhibition of the RhoA/Rho-kinase (ROCK) signalling pathway in vascular smooth muscle (Sauzeau et al., 2000), rat coelic artery (Teixeira et al., 2005) and rat aorta (Chitaley and Webb, 2002). On the other hand, the RhoA/Rho-kinase pathway prevents protein kinase B/Akt-dependent eNOS activity in human endothelial cells (Ming et al., 2002). A more recent study has provided evidence that Rho-kinase signalling activity was amplified in endothelial nitric oxide synthase (eNOS) null mice (Williams et al., 2006). Further complexity arises from results suggesting that arachidonic acid generated by phospholipids can activate ROCK (Araki et al., 2001, Guo et al., 2003), and perhaps contribute to Ca^2 +^-sensitization by tryptamine.

#### 4.2.3 Prostacyclin

Prostacyclin (PGI_2_) is a vasodilator prostanoid produced by endothelial cells as a key mediator in the regulation of vascular tone and blood pressure. Thus potentiation of the tryptamine-elicited vasoconstriction by endothelial cell denudation can be partly explained by removal of prostacyclin as well as removal of vasodilator nitric oxide. PGI_2_ exerts its cellular effects by binding to a G protein-coupled receptor, IP. Stimulation of the IP receptor, coupled to G_s_-type G protein, activates adenylate cyclase leading to cAMP formation, and therefore to vasodilatation of the mesenteric vessels (Hata and Breyer, 2004). We examined whether tryptamine would release prostacyclin by use of a potent prostacyclin synthase antagonist, tranylcypromine (Xavier et al., 2008). The vasoconstrictor response was augmented at the lower doses of tryptamine, suggesting that at lower concentrations prostacyclin was indeed released by tryptamine. However, tranylcypromine is also a non-selective inhibitor of monoamine oxidases (MAO) A and B (Blackwell, 1963). MAO A and B activities appear to be associated with the mesenteric arteries of various species (Coquil et al., 1973, Caramona, 1982). Thus, an alternative explanation for the enhanced responses at lower doses is that tryptamine is metabolised by MAO in the mesenteric bed and its inhibition by tranylcypromine allows elevated levels to reach the receptors.

### 4.3 Monoamine transporters

Cocaine is a nonselective, competitive inhibitor of monoamine reuptake, inhibiting the dopamine (DAT), noradrenaline (NAT) and 5-HT (serotonin, SERT) transporters with K_i_ values of 267 nM, 872 nM and 392 nM, respectively (Torres et al., 2003). Therefore, cocaine is over 2-fold more potent at the serotonin than the noradrenaline transporter. Tryptamine is a substrate for the serotonin transporter (Segonzac et al., 1985, Adkins et al., 2001) and mesenteric arteries are known to express the SERT (Ni et al., 2004). Thus, inhibition of the transporter would be expected to increase the tryptamine concentration in mesenteric circulation. Indeed, cocaine moderately potentiated the contractile response of mesenteric arteries to tryptamine. When cocaine was combined with l-NAME, there was an additional potentiation which we assume is due to the combined additive effects of inhibition of tryptamine transport and removal of the opposing vasodilator effects of released NO. There is one recent report of regulation of nitric oxide enhancing the activity of the noradrenaline transporter to control blood pressure responses to tyramine in anaesthetized rats (Simaan and Sabra, 2011). However, this does not appear to apply to tryptamine responses here, as l-NAME would have opposed the action of cocaine rather than enhancing it.

### 4.4 Summary

In summary, tryptamine causes vasoconstriction of rat mesenteric arterial beds which is mediated via 5-HT_2A_ receptors. This response is due to a coupling between the tryptaminergic receptors, phospholipases C and A_2_ and contractile prostaglandins (PGE_2_). Signalling through the RhoA/ROCK pathway is also implicated. There is a simultaneous release of vasodilator nitric oxide and possibly prostacyclin from the endothelium which oppose and homeostatically balance the increases in pressure. Thus, circulating levels of tryptamine derived from endogenous synthesis or from dietary intake can exert a regulatory control of mesenteric blood flow and thus the digestive and absorptive activities of the gastrointestinal tract.

## Conflict of interest disclosure

The authors declare no conflicts of interest.
